# Rice plants reduce methane emissions in high-emitting paddies

**DOI:** 10.12688/f1000research.15859.3

**Published:** 2019-07-25

**Authors:** Masato Oda, Nguyen Huu Chiem

**Affiliations:** 1 Crop, Livestock and Environment Division, Japan International Research Center for Agricultural Sciences, Tsukuba, 305-8686, Japan; 2 Department of Environmental Science, College of Environment and Natural Resources, Can Tho University, Can Tho, Vietnam

**Keywords:** Greenhouse gases, Mekong Delta, Methane oxidation, Methanogenesis inhibition, Rice paddy, Triple cropping

## Abstract

**Background:** Rice is understood to enhance methane emissions from paddy fields in IPCC guidelines. However, rice actually has two opposite functions related to methane: i) emission enhancement, such as by providing emission pathways (aerenchyma) and methanogenetic substrates; and ii) emission suppression by providing oxygen pathways, which suppress methanogenesis or enhance methane oxidation. The overall role of rice is thus determined by the balance between its enhancing and suppressing functions. Although previous studies have suggested that rice enhances total methane emissions, we aimed to demonstrate in high-emitting paddy fields that the overall methane emission is decreased by rice plants.

**Methods:** We compared methane emissions with and without rice plants in triple cropping rice paddy fields in the Mekong Delta, Vietnam. The gas samples are collected using chamber method and ware analyzed by gas chromatography.

**Results:** We found that rice, in fact, suppressed overall methane emissions in high-emitting paddies. The emission reductions increased with the growth of rice to the maximum tillering stage, then decreased after the heading stage, and finally recovered.

**Discussion**:  Our result indicates that the overall methane emission is larger than that of rice planted area. In addition, although many studies in standard-emitting paddies have found that the contribution of soil organic matter to methanogenesis is small, prior studies in high-emitting paddies suggest that methanogenesis depended mainly on soil organic matter accumulated from past crops. The higher the methane emission level, the lower the contribution of the rice-derived substrate; conversely, the higher the contribution of the rice providing oxygen. Finally, rice plants reduce methane emissions in high-emitting paddies.

**Conclusion:** The present study demonstrates that during the growing season, rice is suppressing methane emissions in high-emitting paddies. This means the significance of using the rice variety which has high suppressing performance in high-emitting paddies.

## Introduction

The role of rice in methane (CH _4_) emissions changes according to emission levels. Because, rice performs three key functions related to CH _4_ emissions: i) providing a CH _4_ pathway through a well-developed system of intercellular air spaces (aerenchyma), ii) providing a substrate for methanogenesis, and iii) oxidizing CH _4_ in rhizosphere by supporting O _2_ counter-transport through aerenchyma system ^1– 6^. The level of contribution of these functions varies with the overall emissions, and the total amount of CH _4_ emitted to the atmosphere is thus a balance between CH _4_ production and oxidation ^6^. To the best of our knowledge, rice enhances overall CH _4_ emissions from paddy fields. In addition, previous studies have mostly disregarded a potential impact of overall emission levels on the role of rice in enhancing or suppressing CH _4 _emissions.

Cicerone and Shetter measured CH _4_ emissions with a closed chamber on the water surface of a paddy field, and found that 4 hours after starting measurement, CH _4_ concentration was 290 ppm over rice plants and only 4 ppm over open water ^1^. Further studies have revealed that CH _4_ produced under methanogenesis diffuses through the soil, which is oxidized by the surface barrier before reaching the atmosphere ^2, 7^. Rice absorbs diffused CH _4_ from its roots and emits CH _4_ through aerenchyma ^3, 5, 7^. These facts suggest that CH _4_ is not emitted from the soil without rice. Other recent studies have provided additional evidence that the primary source of CH _4_ is current-season photosynthates—specifically, root exudates or decaying tissues ^8– 11^. This results in CH _4_ emissions that peak during the late stage of rice growth. Thus, the presence of rice plants has been determined to be the cause of CH _4_ emissions in paddy fields.

Wassmann *et al.* ^12^ measured CH _4_ emissions on the water surface of a paddy field amended with organic matter. They found that organic matter incorporation increased total CH _4_ emission levels from 27–90 to 160–240 kg CH _4_ ha ^−1^ crop ^−1^, and the direct emission from soil by ebullition increased from 15–23% to 35–62%, respectively ^12^. Since it is known that organic matter incorporation causes CH _4_ emissions to peak during the early stages of rice growth ^12^, when the rice is still small and the aerenchyma is not well developed, the results of Wassmann *et al.* ^12^ should be closely examined to determine whether ebullition increased with total emissions. According to the 2006 Intergovernmental Panel on Climate Change (IPCC) guidelines, CH _4_ emissions for 100 days of rice cropping are 130 kg CH _4_ ha ^−1^ crop ^−1^; however, emissions were observed at almost twice this value by Wassmann *et al.* ^12^ Average CH _4_ emissions from rice paddies in Asia without and with organic matter incorporation ranged from 16 to 200 and 250 to 500, respectively ^13^. Although ebullition has been little studied ^14^, we think ebullition must occur at the high emission levels. Furthermore, in the study by Wassmann *et al.* ^12^, twin CH _4_ emissions peaks appeared, with an early peak corresponding to the organic matter amendment and a later peak corresponding to rice-originated substrate ^12^. An alternate interpretation of these results is that the twin peaks could reflect the oxidation performance of rice, since CH _4_ oxidation is known to increase with rice growth, up to the maximum tillering stage, and then decrease ^15^.

We monitored CH _4_ emissions in the paddy fields for 5 years (total 15 crops) in triple rice cropping fields in the Mekong Delta, Vietnam. The CH _4_ emission level was an order of magnitude higher than IPCC standards. These high CH _4_ emissions suggest that ebullition must have been occurring. We hypothesized that rice plants decrease overall methane emission based on the extremely high emission levels. It will be epochal if present the fact that rice plants decrease overall methane emission in paddy fields; because, rice is believed enhancing overall CH _4_ emissions from paddy fields.

## Results and discussion

**Figure 1.**
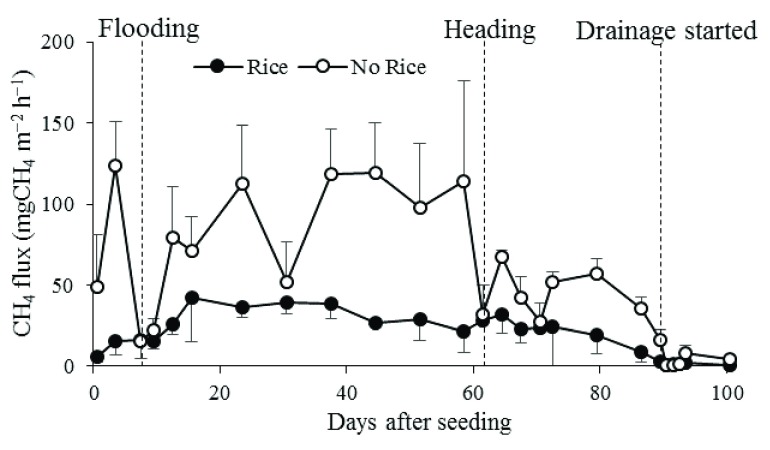
Seasonal CH _4_ emissions in paddy fields. Average CH _4_ emissions of rice-planted areas and no-rice-planted areas in triple cropping rice fields in the Mekong Delta. Winter–Spring season (2017). The paddy fields did not receive rice straw incorporation. Error bars are s.d. (n = 3).

**Figure 2.**
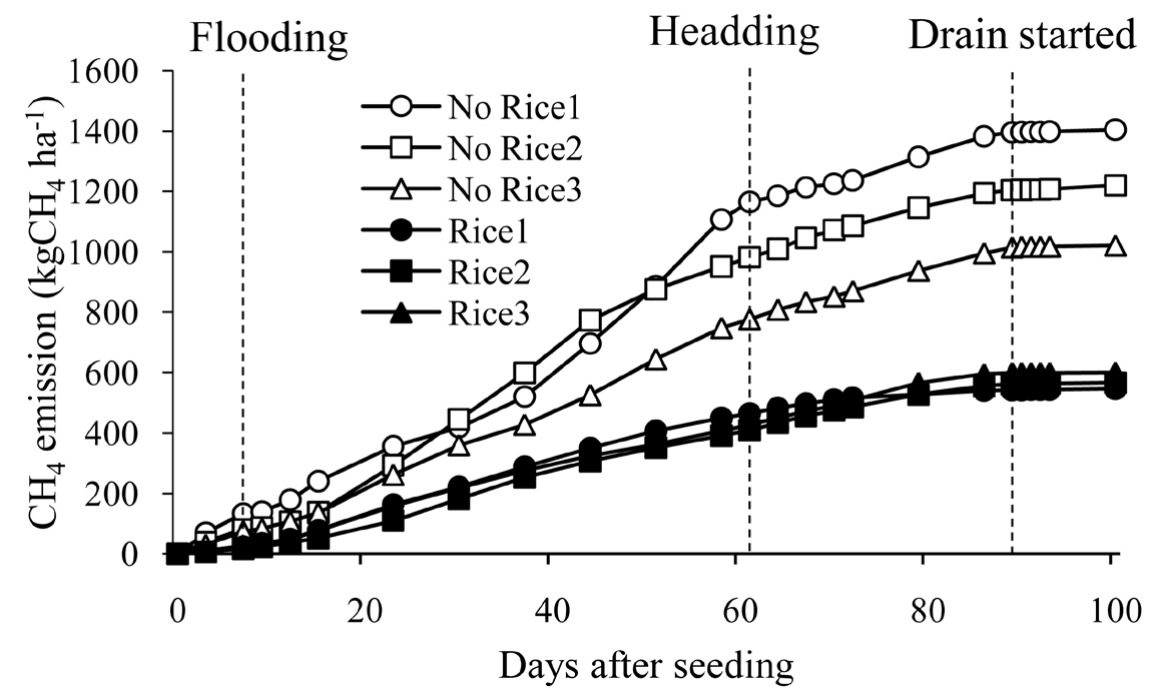
Cumulative CH _4_ emissions in paddy fields. Cumulated CH _4_ emissions of rice-planted areas and no-rice-planted areas in triple cropping rice fields in the Mekong Delta. The number in the legend relates to the fields.

We compared CH _4_ emissions with (Rice) and without rice (No Rice) on the water (soil) surface of triple cropping rice paddy fields in the Mekong Delta. Our results showed that rice presence decreased CH _4_ emissions by half of No Rice. The effect of rice was large even in the early growth stage because of the high plant density (230 kg ha ^−1^ in dry weight; approximately 3 cm interval) and the rapid growth in the tropical climate (see Figure 1, Figure 2). Although the high CH _4_ emission is shown in the first week, it is considered to be caused by the erratic character of ebullition. In fact, there was no difference in the emission between Rice and No Rice on both the 7th and 9th day (see Figure 1). The difference of the total emissions between the treatments is significant (p=0.013; one-sided Welch’s t-test). More importantly, the treatments have formed each group ( Figure 2). This supports the difference caused by not the locations but the treatments. The p-value is 0.013 if the difference made by location. Complete, unprocessed data are available on figshare ^16^. There was no marked CH _4_ emissions peak in the late-stage of the rice mentioned in previous studies ^8– 11^. This suggests that the amount of methanogenesis from the rice-derived substrate is relatively small. Note the high emission levels (500–1400 kg CH _4_ ha ^−1^ crop ^−1^). These findings suggest that total CH _4_ emissions are reduced by oxidation or methanogenesis inhibition associated with growing the rice plant.

We also found that the reduction rate of CH _4_ emissions increased with the growth of the rice plant. The CH _4_ reduction rate was calculated using a moving average of five values by the following formula.

reduction rate = (No Rice – Rice)/No Rice

**Figure 3.**
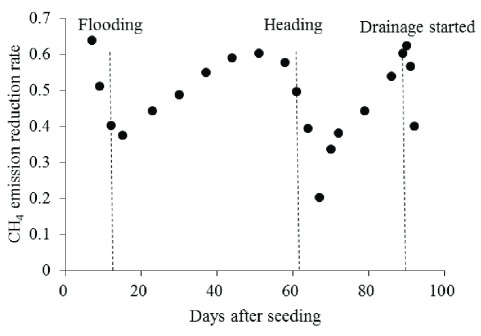
Reduction rate of CH _4_ emissions. The CH _4_ reduction rate was calculated by (No Rice – Rice)/No Rice.

The rate peaked at maximum tillering stage, then bottomed at after heading stage, and then recovered (see Figure 3). The decrease around the heading stage was caused partially by an increase of emissions in rice-planted areas, and mainly by a decrease of emissions in the unplanted area.

No consensus has yet been reached on the extent to which methanotrophs or rice roots attenuate CH _4_ emissions ^17^. Using the N _2_ atmosphere technique, CH _4_ oxidation ratios have been found to be around 40% on average and were relatively stable throughout the rice growing season ^17^. However, genuine CH _4_ oxidation, as measured using inhibitors, tend to decrease with rice growth, and the reduction rate for total CH _4_ emissions can reach up to 20% ^17^. Although, most of those studies assume plant-mediated transportation ^17^, in general, our results roughly matched the results using the N _2_ atmosphere technique. This suggests that the reduction in CH _4_ emissions is not due to genuine oxidation and is more likely to be due to methanogenesis inhibition by oxygen from aerenchyma, which lasts until harvesting ^18^.

We found high CH _4_ emissions in unplanted paddy fields of which not incorporate organic materials. Despite this lower input of methanogenesis substrate, CH _4_ emission levels were 12 times higher than the IPCC guidelines. The emission levels remained almost stable after reaching a maximum. This suggests that methanogenesis at our site mainly depends on soil organic matter that has been accumulated from past rice crops. Prior studies have suggested that the contribution of soil organic matter to methanogenesis is small; however, these studies also indicated that higher emission levels tend to be associated with higher contribution rates of soil organic matter ^8– 11, 19^. Furthermore, a recent study found that large rice plants reduce CH _4_ emissions compared to small rice plants in paddy fields with high soil C contents; instead, they show the opposite effect in paddies with low soil C contents ^20^. Thus, our results are consistent with prior studies that assume that emission levels are proportional to the amount of soil organic matter which can be a methanogenesis substrate.

High-emitting paddies of CH _4_ emissions, which exist widely across tropical Asia ^13^, would have substantial soil organic matter stock formed by sequential rice cropping under flooded conditions ^21^. For instance, the use of a rice variety which has better performance of methanogenesis inhibition in high-emitting paddies is very effective; the 10 % reduction is equivalent to 100% of methane emission in standard paddy fields. On the other hand, without rice plants, the methane emission from the existing soil organic matter stock will double by ebullition; the 100% increase is equivalent to 1000% of the standard paddies. This suggests that the future study for the soil organic matter stock map is critical.

## Conclusion

To our knowledge, most studies of CH _4_ emissions in paddy fields have been conducted in fields with low overall emission levels. Since the role of rice in CH _4_ emissions varies according to the overall emission levels, these results cannot be appropriately generalized to rice paddies with high emission levels. The results of our study suggest that rice reduces emissions in high-emitting paddies. This means the significance of using the rice variety which has high suppressing performance in high-emitting paddies. The emission levels are related to the amount of soil organic matter which can be a methanogenesis substrate; this suggests that the future study for the soil organic matter stock map is critical.

## Methods

### Study site

**Figure 4.**
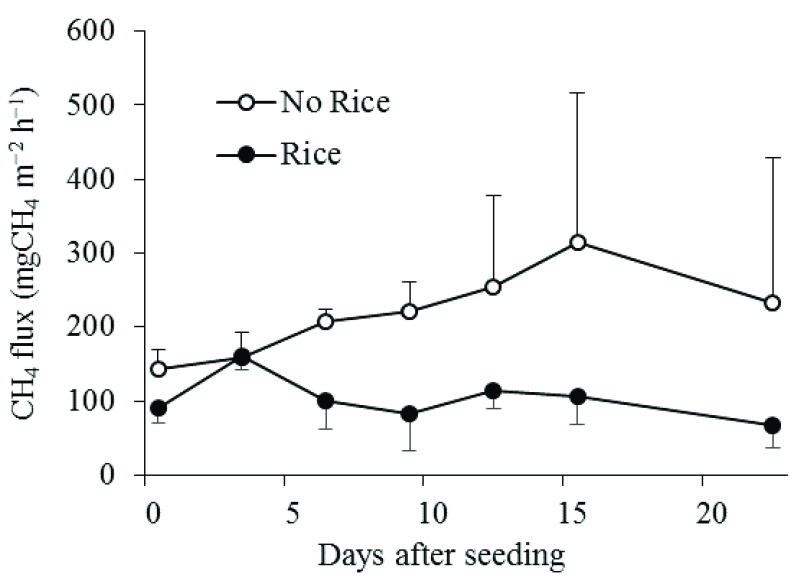
Seasonal CH _4_ emission in paddy fields. Average CH _4_ emissions of rice-planted areas and no-rice-planted areas in triple cropping rice fields in the Mekong Delta. Summer–Autumn season (2016). The paddy fields received incorporated rice straw of the previous crop. Error bars are s.d. (n = 3).

Experimental fields were in Tan Loi 2 Hamlet, Thuan Hung village, Thot Not district, Can Tho city, Vietnam. Farmers conduct triple rice cropping by direct seeding and full flooding. This district receives almost 2 months of a flood annually from the Mekong River. The flood decomposes rice straw underwater to the extent that it is no obstacle for seeding. Therefore, farmers start the rice cropping by leveling the fields, without incorporating rice straw. We observed CH _4_ emissions in three paddy fields (26 × 17 m each) under conventional conditions for 5 years from September 2011. A preliminary study was conducted with the rice variety OM501 (suitable for the season) in the Summer-Autumn season of 2016 by the same methods and paddies of the present study (see Figure 4). In the present study, we used the three fields for replication. We conducted the present study after the annual flood (4 November 2016–12 February 2017); these fields did not incorporate rice straw because of the period was after the annual flood.

### Treatment

We compared CH _4_ emissions with (Rice) and without rice (No Rice). We set 2 × 2 m squares of plastic films on a part of each three fields just before seeding, then carefully removed them with seeds on the films immediately after seeding. In other points, there was no difference with farmers’ conventional rice-growing procedures. Farmers scattered 230 kg ha ^−1^ (in dry weight) of germinated rice seed (variety Jasmine) on drained wet paddy fields’ surfaces on 5th November. This wet condition was maintained for 7 days, then the fields were kept flooded until 89 days after seeding (DAS), and the rice harvested on 100 DAS. The farmers applied fertilizer, which included 76 kg of urea on 12 November, 53 kg each of urea and NPKS (16-16-8-13), diammonium phosphate on 19 November, and 53 kg each of urea and NPKS on 15 December. The daily average water levels were monitored with water level loggers (HOBO U20; Onset Computer Corporation, Bourne, Massachusetts) at the corner of the fields, and the average levels were 2.0 cm (−0.6 to 6.1 cm) until drained.

### Measurement of CH _4_ emissions

We set an approximately 2 m long and 0.5 m wide ladders from the center of the shorter bund to allow measurement of CH _4_ without touching the paddy soil surface. Those ladders were on the borders of the non-planted areas in each field. We set PVC chamber bases on the paddy fields of both sides of the ladders to avoid measurement perturbation. Chambers (60 × 80 cm and 100 cm high, transparent acryl) were set on a watertight chamber bases for every measurement. Measurements were taken at 8 a.m. because previous research has indicated that emissions at this time have a high correlation (ca. 90% of average emission) with average daily emissions ^22^. We mixed the air in the chamber with a fan for 5 min after setting the chamber, then sampled the first gas, then sampled the second gas 20 min later. We conducted the measurements once a week throughout the rice growing stage, but every 3 days for 2 weeks after seeding, heading stage, and around draining. The samples were analyzed by gas chromatography (GC-14B, Shimazu, Kyoto). The cumulative CH _4_ emissions were calculated by linear interpolation.

### Ethics statement

This study was conducted with the approval of the farmer.

### Statistical analysis

Data were processed using Microsoft Excel 2016.

## Data availability

Raw data of this article is available from figshare: https://doi.org/10.6084/m9.figshare.6916277.v1 ^16^. Data are available under the terms of the Creative Commons Zero “No rights reserved” data waiver (CC0 1.0 Public domain dedication).
