# Supplementary material for: Rice plants reduce methane emissions in high-emitting paddies
Source: F1000Res. 2019 Jul 25;7:1349. Originally published 2018 Aug 29. [Version 3] doi: 10.12688/f1000research.15859.3 (PMC6659764; doi:10.12688/f1000research.15859.3)
Supplement: Peer review history

# Peer review: Reviewer response for version 3

**DOI:** 10.5256/f1000research.22008.r51633

**Published:** 2019-08-15

**Authors:** Kees Jan van Groenigen

**Affiliations:** 1 Department of Geography, College of Life and Environmental Sciences, University of Exeter, Exeter, UK

The manuscript has further improved and it seems scientifically sound. Based on these standards, the manuscript can be accepted for indexing. However, the quality of the writing still leaves something to be desired; the text is quite hard to follow at times and it still contains several grammatical errors. I've made some suggestions before, but I don't have time to point out all grammatical errors in each new version.

I confirm that I have read this submission and believe that I have an appropriate level of expertise to confirm that it is of an acceptable scientific standard.

# Peer review: Reviewer response for version 3

**DOI:** 10.5256/f1000research.22008.r51631

**Published:** 2019-07-25

**Authors:** Dung Duc Tran

**Affiliations:** 1 Center of Water Management and Climate Change (WACC), Vietnam National University – Ho Chi Minh City (VNU – HCM), Ho Chi Minh City, Vietnam

The authors have revised the manuscript based on my comments. It is now accepted for indexing.

 Thanks.

I confirm that I have read this submission and believe that I have an appropriate level of expertise to confirm that it is of an acceptable scientific standard.

# Peer review: Reviewer response for version 2

**DOI:** 10.5256/f1000research.21593.r50479

**Published:** 2019-07-09

**Authors:** Dung Duc Tran

**Affiliations:** 1 Center of Water Management and Climate Change (WACC), Vietnam National University – Ho Chi Minh City (VNU – HCM), Ho Chi Minh City, Vietnam

The manuscript has been improved based on the comments. However, I recommended the authors to consider the following 2 points before approval for indexing:

1. The authors replied: "We corrected the method section and clarified the details. For the site location map, we think it can be found on the internet nowadays.". I do not agree, please add the Figure because the common audience doesn’t have much time to find the location.
2. Conclusion is not quite sufficient in terms of relating to previous findings.

I confirm that I have read this submission and believe that I have an appropriate level of expertise to confirm that it is of an acceptable scientific standard, however I have significant reservations, as outlined above.

# Response

**Published:** 2019-07-22

**Authors:** Masato Oda

**Affiliations:** Japan International Research Center for Agricultural Sciences (JIRCAS), Japan

Thank you for the comment.

**1. For the site local map:**

We added the link to the Google map according to the article guidelines of F1000Research. The link takes no time for the reader of F1000Research, in addition, it’s scalable.

**2. Conclusion:**

We added a conclusion which was presented in the abstract but not presented in Conclusion sections. The aim of the present study is the role of rice in the paddy field for methane emission in the historical context. Therefore, we mentioned it in the first sentence of the abstract as “Rice is understood to enhance methane emissions from paddy fields”, then concluded it in the Conclusion as “To our knowledge, most studies of CH 4 emissions in paddy fields have been conducted in fields with low overall emission levels. Since the role of rice in CH 4 emission varies according to the overall emission levels, these results cannot be appropriately generalized to rice paddies with high emission levels.”

# Peer review: Reviewer response for version 2

**DOI:** 10.5256/f1000research.21593.r50481

**Published:** 2019-07-05

**Authors:** Azeem Tariq

**Affiliations:** 1 UMR 951 Innovation SupAgro-INRA-CIRAD, Montpellier SupAgro-IRC, Montpellier, France

I have checked the authors’ report. It is hard to follow the changes made by authors in the new version. Further, the authors have not justified all the points raised in my first report.

I confirm that I have read this submission and believe that I have an appropriate level of expertise to confirm that it is of an acceptable scientific standard, however I have significant reservations, as outlined above.

# Response

**Published:** 2019-07-22

**Authors:** Masato Oda

**Affiliations:** Japan International Research Center for Agricultural Sciences (JIRCAS), Japan

Thank you for reconsidering our manuscript. The methodology is described in the first sentence of the ‘Results and discussion’: “We compared CH _4_ emissions with (Rice) and without rice (No Rice) on the water (soil) surface of triple cropping rice paddy fields in the Mekong Delta”. Our treatment is only “with” or “without” rice, the other condition is the same as farmers’ conventional. See the second sentence in the ‘treatment’ of ‘Methodology’ section: “We set 2 × 2 m squares of plastic films on a part of each three fields just before seeding, then carefully removed them with seeds on the films immediately after seeding. In other points, there was no difference in farmers’ conventional rice-growing procedures.”

Other responses are as follows:

**1. Water management:**

The water management is full flooding. See the second sentence of the ‘Methods’ section: “Farmers conduct triple rice cropping by direct seeding and full flooding.”

**2. Justification of the first-week emission:**

See the third sentence of the ‘Results and discussion’: “Although the high CH _4_ emission is shown in the first week, it is considered to be caused by the erratic character of ebullition. In fact, there was no difference in the emission between Rice and No Rice on both the 7th and 9th day (see Figure 1).” Furthermore, we added the following sentence: “The difference of the total emissions between the treatments is significant (p=0.013; one-sided Welch's t-test). More importantly, the treatments have formed each group (Figure 2). This supports the difference caused by not the locations but the treatments. The p-value is 0.013 if the difference made by location.” In addition, supplemental figure 4 is the supporting data.

**3. Input straw:**

Both no input. See the fourth sentence of the ‘Methods’ section: “The flood decomposes rice straw underwater to the extent that it is no obstacle for seeding. Therefore, farmers start the rice cropping by leveling the fields, without incorporating rice straw.”

**4. Conclusion section:**

You can see the conclusion section.

# Peer review: Reviewer response for version 2

**DOI:** 10.5256/f1000research.21593.r50480

**Published:** 2019-07-04

**Authors:** Kees Jan van Groenigen

**Affiliations:** 1 Department of Geography, College of Life and Environmental Sciences, University of Exeter, Exeter, UK

While this revised version has improved, I still have some issues with this manuscript. Importantly, the comments by reviewer 1 weren't all fully addressed. Moreover, the manuscript contains *many *statements that are not supported by any references, and ideas that are not adequately explained. For instance:

- 2nd to last sentence of the introduction: Who is "our"?  Your paper tries to prove the opposite, so "our" is not "we". Do you mean "It is generally assumed that rice enhances overall CH4 emissions from paddy fields."? In that case, please add a reference to support this statement.
- 2nd paragraph of introduction: "Therefore, an established theory has emerged that CH4 is not emitted from the soil without rice." This statement needs a reference.
- Page 3, top of right column. First time you mention “ebullition”. The text hasn't explained yet what this means, and how important this process is for overall CH4 emissions.
- Page 3, top of right column: "Since it is known that organic matter incorporation causes CH4 emissions to peak during the early stages of rice growth". Reference needed.
- Page 3, right column: "Although ebullition has been little studied, ebullition must occur at high emission levels." Why? Please explain and provide references.
- Page 5, left column: "High-emitting paddies of CH4 emissions, which exist widely across tropical Asia…". Reference needed.
- First sentence of the conclusion: "Most studies of CH4 emissions in paddy fields have been conducted in fields with low overall emission levels." Again, no reference provided. A reference to a review article showing that this is true would help.

The text is unclear in several places. For instance:

- Abstract: "our results in high-emitting paddies suggest that methanogenesis depended mainly on soil organic matter accumulated from past crops." How did you come to this conclusion?
- Abstract: "The higher the methane emission level, the lower the contribution of the rice-derived substrate". How does this interpretation follow from your own results?
- Last sentence of introduction: "It will be epochal if present the fact that rice plants decrease overall methane emission in paddy fields." The meaning of this sentence is unclear. 
- Results, line 9: Why is "ebullition" between quotation marks?
- Page 4, left column, top paragraph: "The decrease around the heading stage was caused partially by an increase of emissions in rice-planted areas, and mainly by the erratic emissions in the unplanted area." If this decrease is erratic, then how can we be sure that the high emissions earlier in the growing season aren't erratic as well? How can we be sure that high emissions before planting in the unplanted plots are not erratic?
- Page 5, top of right column: "We observed CH4 emissions in 18 paddy fields (26 × 17 m each) under several conditions for 5 years from September 2011." Where are these data reported? If you don't report them, then why mention this? The earliest data that are reported in this manuscript are from 2016.
- Page 5, top of right column: "In the present study, we used three fields for replication." Are these fields the same fields that were described above (i.e. 26*17 m in size)? This is not clear.

The article still contains quite a few grammatical errors or poor structure. A few suggestions:

- Abstract, methods section: Delete "of" to write "methane emissions with and without rice plants".
- Last sentence of intro: "We hypothesised that rice plants decrease overall methane emission based on the extremely high emission levels."
- First sentence of the results section would be a good place to mention study location.
- 2nd sentence of results: "Our results showed that rice presence decreased CH4 emissions by half."
- Page 4, right column: "This suggests that methanogenesis at our site mainly depends on soil organic matter that has been accumulated from past rice crops."
- Last paragraph of results section: Change "huge" to "large", or "substantial", and provide references to support this statement.

I confirm that I have read this submission and believe that I have an appropriate level of expertise to confirm that it is of an acceptable scientific standard, however I have significant reservations, as outlined above.

# Response

**Published:** 2019-07-22

**Authors:** Masato Oda

**Affiliations:** Japan International Research Center for Agricultural Sciences (JIRCAS), Japan

Thank you very much for your kind suggestion. Furthermore, thank you so much for correcting and improving the English. We completed the lack of references.

We revised the manuscript for the other points as follows:

**1.   Page 3: The explanation of “ebullition”.**

We added the words "the direct emission from soil by".

**2.   Introduction: The reason of epochal.**

We added as follows: “; because, rice is believed enhancing overall CH _4_ emissions from paddy fields”.

**3.   Abstract: The reason of the methanogenesis source.**

We correct as follows: “our results” to “prior studies”.

**4.   Abstract: “The higher…How does this interpretation...?”**

Thank you for leading. We changed the last part as follows: “; conversely, the higher the contribution of the rice providing oxygen. Finally, rice plants reduce methane emissions in high-emitting paddies.”

**5.   Results, line 9: Why is "ebullition" between quotation marks?**

We deleted.

**6.   Page 4, left column, top paragraph: The explanation of erratic.**

We changed to “a decrease of”.

**7.   Page 5, top of right column: 18 paddy fields…**

We corrected “18” to “3”, and “several” to “conventional”.

**8.   Page 5, top of right column: The field size.**

It is the same size because of the same fields. We think it is obvious by the above correction for the number of fields.

**9.   To mention the study location in the results section.**

# Peer review: Reviewer response for version 1

**DOI:** 10.5256/f1000research.17312.r48479

**Published:** 2019-06-14

**Authors:** Dung Duc Tran

**Affiliations:** 1 Center of Water Management and Climate Change (WACC), Vietnam National University – Ho Chi Minh City (VNU – HCM), Ho Chi Minh City, Vietnam

The authors of "Rice cultivation reduces methane emissions in high-emitting paddies" present an interesting manuscript with great effort to demonstrate the balance between rice’s emitting and suppressing function changes according to overall methane emission levels. In general, the manuscript is well-written. However, I recommend the authors to do a major revision to the manuscript before being potentially accepted for indexing by the Journal.

1. The Introduction section should add research objectives or in terms of research questions that help readers to promptly understand the paper's content.
2. In the Methods section, description and activities are needed in more detail; additionally a map of the case study would be useful.
3. In the Results and Discussion section, I expected more discussion that enhance more work on comparing findings with previous studies. In addition, the limitations and future outlooks need to be elaborated upon.
4. A Conclusion is important to add into the manuscript since it presents the real implication of the research based on the authors' perspective.

I confirm that I have read this submission and believe that I have an appropriate level of expertise to confirm that it is of an acceptable scientific standard, however I have significant reservations, as outlined above.

# Response

**Published:** 2019-06-17

**Authors:** Masato Oda

**Affiliations:** Japan International Research Center for Agricultural Sciences (JIRCAS), Japan

Thank you very much for your precious suggestions. 

1. We improved the abstract and the introduction to describe more clearly the aim of this study. 

2. We corrected the method section and clarified the details. For the site location map, we think it can be found on the internet nowadays. 

3. We added the latest reference which is expected to give readers the deeper understanding of our findings to the result and discussion section. 

4. For the limitations and future outlooks, we described them at the end of the discussion section and the newly added conclusion section.

See the revised manuscript, please.

# Peer review: Reviewer response for version 1

**DOI:** 10.5256/f1000research.17312.r46457

**Published:** 2019-05-28

**Authors:** Kees Jan van Groenigen

**Affiliations:** 1 Department of Geography, College of Life and Environmental Sciences, University of Exeter, Exeter, UK

Oda and Chiem present CH _4_ fluxes from Vietnamese rice paddies with- and without rice plants, and conclude that the presence of rice plants reduces CH _4_ emissions. Their results are interesting and convincing, and the experimental design seems solid. That said, I have several serious reservations about this paper that need to be addressed before it can be approved. 

1.  The authors need to explain more clearly the relevance of their research.  It might be true that the presence of rice plants reduces CH _4_ emissions, but the *sole purpose* of paddies is to support rice agriculture. Thus, the statement that rice cultivation reduces methane emissions from paddies seems a bit strange. Does the IPCC provide estimates of emissions from rice paddies without rice plants? If not, how exactly would the results from this study contribute to understanding the contribution of rice agriculture to CH _4_ emissions?
2. The experimental design does not allow the authors to test their hypothesis as stated in the abstract. The authors aim to demonstrate that the effect of rice plants on methane emissions depends on the background emissions (i.e. without plants). However, the authors only test the effect of rice plants in paddies with high background emissions.
3. The authors did not test the significance of differences between plots with- and without rice plants.
4. The reference list is quite outdated; only 2 of the cited studies were published in the last 5 years. Several relevant recent papers are ignored by the authors. For instance, Jiang *et al*. 2017 ^1^ reported that in paddies with high soil C contents, large rice plants reduce CH _4_ emissions compared to small rice plants. Furthermore, they show the *opposite* effect in paddies with low soil C contents. Please note that I am a co-author on that paper; I don’t suggest that the authors need to include it, but please consider this as just one example of relevant recent research.
5. The first sentence of the introduction is essentially an hypothesis. Yet, the authors provide no clear support to back up this statement.
6. In the last sentence of the introduction, what does “all studies” refer to? The studies 1-6? Did these studies compare CH _4_ emissions with and without rice plants? This is not clear.
7. The text is slightly confusing is some places. For instance, in the “background” section in the abstract it’s not clear whether the authors refer to methane emissions with or without rice plants. The same is true for the last paragraph of the introduction. On page 4, second paragraph, the discussion of changes in “reduction rates” is quite confusing. Does a decrease in reduction rates mean means relatively low or high rates in plots with plants?
8. Some basic info about the field site is missing. For instance, MAT and MAP data would be useful, as well as soil C contents.
9. I spotted a few grammatical errors. For instance, in the background section of the abstract, “existing studies” should be “previous studies”. In the discussion section of the abstract “rice-providing” should be “rice-derived”. The first sentence of the results and discussion section should read “In the present study, we compared CH _4_ emissions on the water (soil) surface of paddy fields with and without rice”. These are just a couple of examples. Perhaps the authors could contact a native speaker to have a quick look at their paper?

I confirm that I have read this submission and believe that I have an appropriate level of expertise to confirm that it is of an acceptable scientific standard, however I have significant reservations, as outlined above.

## References

[1] : Higher yields and lower methane emissions with new rice cultivars. *Glob Chang Biol*.23(11) : 10.1111/gcb.13737 4728-4738 10.1111/gcb.13737 28464384

# Response

**Published:** 2019-06-14

**Authors:** Masato Oda

**Affiliations:** Japan International Research Center for Agricultural Sciences (JIRCAS), Japan

1. Thank you for your precious suggestion! We changed the word "cultivation" to "plants" in the title. We think the word is more suitable to the content.
2. Thank you again. We aim to demonstrate rice plants are suppressing overall methane emission in high CH4 emission paddy fields. We changed the last sentence of the background as follows. "that the balance..." to "that the overall methane emission is decreased by rice plants." We expect that this change clarifies some other points that you indicate. In association with, we think the experiment in the low background is not necessary because they have been conducted by plenty of studies.
3. We think it's obvious from Figure 1 and 2; for your information, the p-value by one-sided Welch's t-test for the total emissions is 0.013.
4. Thank you very much for introducing the study to support our conclusion. We referred to that in the last paragraph of the discussion. We have referred to rather old-dated studies because we aim to mention the role of rice in the context of the history of the study for paddy fields CH4 emission.
5. The sentence indicates common sense; therefore, we would like to change as follows: "Rice is understood to enhance methane emissions from paddy fields as mentioned in IPCC guidelines."
6. We would like to change the sentence ", all studies ... these studies". to "rice enhances overall CH4 emissions from paddy fields. In addition, previous studies".
7. We added the formula, and we clarified the sentences in the results section.
8. We think the soil carbon contents is important; however, that is out of range of this study and we don’t have the map.
9. Thank you so much! We had used an English editing service, but that was not enough. We have revised the English.

# Peer review: Reviewer response for version 1

**DOI:** 10.5256/f1000research.17312.r39719

**Published:** 2019-05-09

**Authors:** Azeem Tariq

**Affiliations:** 1 UMR 951 Innovation SupAgro-INRA-CIRAD, Montpellier SupAgro-IRC, Montpellier, France

The authors of "Rice cultivation reduces methane emissions in high-emitting paddies" present an effort in collecting and measuring the CH _4_ emissions, in three rice cropping cycles during the 100 days of the field experiment. The overall paper has been written in good English and in a logical manner. The major concern in this manuscript is the methodology and conclusion. The methodology is not clear enough, for example the authors do not make clear the explanation of water treatments in fields with rice and without rice. The authors claimed lower CH _4_ emissions due to absence of rice plants, but the difference in the first week (Fig. 1) after seeding when there will be hardly any significant role of rice seedlings is not justified.

 Furthermore, the methodology section is much confusing with the use of decomposition of straw and addition of straw (study site section). It is hard to follow the inputs of straw in fields with and without rice. The authors clearly need to modify the methodology section to make the description of treatments and to justify the results.

 Further, there are no conclusions drawn from the manuscript. The authors forgot to give the conclusion at the end of manuscript.

 In general, the manuscript is not acceptable in its current condition.

I confirm that I have read this submission and believe that I have an appropriate level of expertise to state that I do not consider it to be of an acceptable scientific standard, for reasons outlined above.

# Response

**Published:** 2019-05-21

**Authors:** Masato Oda

**Affiliations:** Japan International Research Center for Agricultural Sciences (JIRCAS), Japan

Thank you very much for reviewing our manuscript and giving precious comments.

I think your comments can be summarized to three points: “uniformity of conditions”, “the effect of early stage rice plants”, and “the reason of high CH4 emission in the first week”.

1) For the first, we would like to apologize for the incomplete explanation and mistype in the treatment subsection that leads readers to misunderstanding for the above points. The sentences and the correct version are as follows:

    ”We set 2 × 2 m squares of plastic films on each field” → "We set 2 × 2 m squares of plastic films on a part of each three fields”

    “In other plots, there was no difference with farmers’ conventional” → ”In other points, there was no difference with farmers’ conventional”

These corrections will make clear that the experiment was conducted under completely uniformed conditions of three pair. These corrections are also the answer for the suspicion of uniformity of conditions.

2) For the second, we think the effect of rice is large even in the early growth stage of rice because of the high plant density (230 kg ha-1) and the rapid growth of them. We would like to apologize again for mistype of the unit.

3) For the third, we think the high CH4 emission in the first week was caused by the erratic character of “ebullition”. You can check there is no difference between "no rice" and "rice" in the 7th and 9th day’s emission by raw data (https://f1000research.com/articles/7-1349/v1#ref-16).

For straws, this is not a treatment but the conventional management of triple cropping rice cultivation in Mekong Delta. They were decomposed in the natural flooding water and not incorporated into the soil (however, straws were incorporated in the preliminary study).

Following the editorial team’s recommendation, we are waiting for additional peer review reports before starting on any article revisions.
